# Phosphorylated EGFR and PI3K/Akt signaling kinases are expressed in circulating tumor cells of breast cancer patients

**DOI:** 10.1186/bcr2149

**Published:** 2008-09-29

**Authors:** Galatea Kallergi, Sofia Agelaki, Antonia Kalykaki, Christos Stournaras, Dimitris Mavroudis, Vassilis Georgoulias

**Affiliations:** 1 Laboratory of Tumor Cell Biology, School of Medicine, University of Crete, Voutes, Heraklion, 71110, Greece; 2 Department of Medical Oncology, University General Hospital of Heraklion, Voutes, Heraklion, 71110, Greece; 3 Department of Biochemistry, School of Medicine, University of Crete, Voutes, Heraklion 71110, Greece

## Abstract

**Introduction:**

The phosphoinositide-3 kinase (PI3K)/Akt pathway, operating downstream of epidermal growth factor receptor (EGFR) and human epidermal growth factor receptor (HER)2, is implicated in cell migration and survival. EGFR and HER2 are expressed in circulating tumor cells, but the activation status of downstream signaling molecules has not yet been reported.

**Methods:**

To investigate expression levels of EGFR, HER2, PI3K, and Akt in circulating tumor cells, we used peripheral blood mononuclear cells from 32 cytokeratin-19 mRNA-positive patients with early (n = 16) and metastatic (n = 16) breast cancer.

Peripheral blood mononuclear cell cytospins were double stained with cytokeratin antibody along with one of the following: EGFR, phospho-EGFR, HER2, phospho-PI3K, or phospho-Akt antibodies.

**Results:**

EGFR and HER2 were expressed in circulating tumor cells of 38% and 50% patients with early and 44% and 63% patients with metastatic disease, respectively. Interestingly, phospho-PI3K and phospho-Akt expression levels were similar at 88% (14 out of 16) and 81% (13 out of 16), respectively, in circulating tumor cells of patients with early and metastatic disease. Phospho-EGFR was observed in circulating tumor cells of two (33%) early and six (86%) metastatic EGFR-positive patients. Immunomagnetic separation of peripheral blood mononuclear cells, using EpCAM antibody, and subsequent double-staining experiments of circulating tumor cells showed that EGFR was co-expressed with HER2, phospho-Akt and phospho-PI3K kinases, indicating activation of the corresponding survival signaling pathway.

**Conclusions:**

Our findings demonstrate that circulating tumor cells express receptors and activated signaling kinases of the EGFR/HER2/PI3K/Akt pathway, which could be used as targets for their effective elimination.

## Introduction

Circulating tumor cells (CTCs) have been identified in the blood of patients bearing a wide range of malignancies [1,2], but not in healthy individuals or in patients with nonmalignant diseases [1]. CTCs have also been identified in significant proportions of patients with both early and metastatic breast cancer, and their presence carries significant prognostic information [3,4]. Indeed, the detection of CTCs before adjuvant chemotherapy as well as the persistence of CTCs after the completion of systemic adjuvant treatment is associated with an unfavorable clinical outcome [4-6]. Similarly, in patients with metastatic disease, elevated CTC numbers before or soon after the initiation of chemotherapy is an indicator of poor prognosis [7,8].

The malignant nature of CTCs is supported by the presence of chromosomal alterations [9-12]. However, it appears that only a small proportion of CTCs are capable of forming overt tumor deposits [13]. The molecular characteristics of these cells may play an important role in their survival and could therefore be used to guide effective treatment strategies.

Epidermal growth factor receptor (EGFR; human epidermal growth factor receptor [HER]1) is a member of the ErbB family of receptors that also includes HER2, HER3, and HER4. EGFR ligand binding induces the formation of homodimers and heterodimers [14,15] and triggers the activation of downstream signaling pathways, such as the phosphoinositide-3 kinase (PI3K)/Akt pathway (among others), which control cell proliferation, survival, and migration [16]. HER2 is the preferred partner for heterodimerization with the other members of ErbB family of receptors [17], and its over-expression has been reported to amplify EGFR signaling [18]. EGFR and ligands such as transforming growth factor-α and amphiregulin are over-expressed in a large subset of primary breast carcinomas [19,20]. Co-expression of these factors in breast cancer confers poor prognosis and resistance to hormonal therapy [21]. Moreover, inappropriate activation [22] or over-expression [23] of EGFR was associated with poor patient outcome.

Recent studies have reported expression of growth factor receptors on CTCs of patients with breast and prostate cancer [15,24-26]. However, little is known about the presence of activated receptors and downstream signaling kinases that regulate pro-survival pathways in the CTCs of breast cancer patients. The objective of this study was to investigate whether EGFR and phosphorylated EGFR are expressed on CTCs isolated from the blood of patients with breast cancer. The expression of HER2 and the activation status of PI3K and Akt kinases operating downstream of EGFR were also evaluated in adjuvant as well as in metastatic settings.

## Materials and methods

### Patient samples and cytospin preparation

A total of 38 patients with detectable cytokeratin (CK)-19 mRNA positive cells [27,28] in peripheral blood were screened using immunofluorescence, and 32 (84.2%) of them with early (n = 16) and metastatic (n = 16) breast cancer who were found to harbor occult tumor cells were enrolled in the study. In addition 20 female normal blood donors were included in the study as negative control individuals. Specifically, peripheral blood (10 ml in EDTA) was obtained before the initiation of adjuvant treatment (usually 3 to 4 weeks after primary surgery) or first-line chemotherapy for metastatic disease. All blood samples were obtained at the middle of vein puncture after the first 5 ml of blood was discarded. These precautions was undertaken in order to avoid contamination of the blood sample with epithelial cells from the skin during sample collection. All patients gave their informed consent to participate in the study, which has been approved by the Ethics and Scientific Committees of our institution.

Peripheral blood mononuclear cells (PBMCs) were isolated with Ficoll-Hypaque density gradient (d = 1,077 g/mol) centrifugation at 1,800 rpm for 30 minutes. The PBMCs were washed three times with phosphate-buffered saline and centrifuged at 1,500 rpm for 10 minutes. Aliquots of 250,000 cells were centrifuged at 2,000 rpm for 2 minutes on glass slides. Cytospins were dried up and stored at -80°C. Four to five slides from each patient were used for staining experiments.

### Cell cultures

For control experiments we used two different breast cancer cell lines, namely MCF7 and SKBR3, which express EGFR, HER2, and the other examined signaling molecules. The SKBR3 breast cancer cell line, which expresses high levels of HER2 and EGFR, was used as a positive control for these molecules, and the MCF7 cell line as a positive control for phosphorylated Akt (pAkt) and phosphorylated PI3K (pPI3K) [29-32]. The MCF7 mammary adenocarcinoma cells (obtained from the American Type Culture Collection, Manassas, VA, USA) were cultured in (vol:vol) 1:1 Dulbecco's modified Eagle medium/Ham's F12 medium (GIBCO-BRL NY, USA), supplemented with 10% fetal bovine serum (GIBCO-BRL NY, USA), 2 mmol/l L-glutamine (GIBCO-BRL NY, USA.), 30 mmol/l NaHCO_3_, 16 ng/ml insulin, and 50 mg/ml penicilline/streptomycin (GIBCO-BRL NY, USA). SKBR3 cells were cultured in McCoy's (GIBCO-BRL Co.), enriched with 10% fetal bovine serum and 2 mmol/l L-glutamine supplemented with 50 mg/ml penicilline/streptomycin.

Cells were maintained in a humidified atmosphere of 5% CO_2_/95% air. Subcultivation for all cell lines was performed with 0.25% trypsin and 5 mmol/l EDTA (GIBCO-BRL Co.). All of the experiments were performed during the logarithmic growth phase. At 15 to 20 hours before the experiments, cells were transferred in serum-starved medium containing only L-glutamine, NaHCO_3_, and penicillin/streptomycin.

### Confocal laser scanning microscopy

The presence of CK-positive cells in PBMCs of cytospin preparations was investigated using two different antibodies, according to the origin of the examined second antibody: the mouse A45-B/B3 (detecting CK8, CK18, and CK19; Micromet, Munich, Germany) for the rabbit anti-EGFR, anti-pEGFR and anti-pAKT, and for the goat anti-pPI3K; and the rabbit anti-pancytokeratin (Santa Cruz, Santa Cruz, CA, USA) for the mouse anti-HER2. The pancytokeratin and HER2 antibodies were validated and their specificity was confirmed in normal donors and in CK-negative patients, as previously reported [26]. Cytospins were also double stained with anti-CD45 (common leukocyte antigen; Santa Cruz, Santa Cruz, CA, USA) and A45-B/B3 antibodies in order to exclude possible ectopic expression of cytokeratins on hematopoietic cells. The cytomorphological criteria proposed by Meng and coworkers [33] (high nuclear/cytoplasmic ratio, larger cells than white blood cells, and so on) were used to characterize a CK-positive cell as a CTC.

Cytospins from the same patients were also stained for EGFR (Santa Cruz, Santa Cruz, CA, USA), pEGFR (Invitrogen, Carlsbad, CA, USA), HER2 (Oncogene, Dermstadt, Germany), pPI3K (Santa Cruz), and pAkt (Cell Signaling, Boston, MA, USA) expressions in double-staining experiments and assessed using confocal laser scanning microscopy [26]. Specific staining can be easily distinguished by double immunofluorescence because of the differential intracellular distribution of the examined molecules compared with nonspecific staining, as reported to Fehm and coworkers [34]. PBMC cytospins were fixed with cold aceton:methanol 9:1 for 20 minutes and stained for cytokeratin with a pancytokeratin antibody, as mentioned above. Subsequently, the same slide was stained with either EGFR, pEGFR, HER2, pPI3K, or pAkt antibodies for 45 minutes. Cells were then incubated with the corresponding secondary antibodies for 45 minutes. Slides were analyzed using a confocal laser scanning microscope module (Leica Lasertechnik, Heidelberg, Germany) and images were analyzed using the respective software.

### Immunomagnetic separation of CTCs

A total of 2 × 10^7 ^PBMCs, isolated as described above, were placed in 1 ml phosphate-buffered saline/20% fetal calf serum. Fifty microliters of CELLection beads (Dynal, Inc., Wirral, UK; coated with EpCAM monoclonal antibody via a DNA linker to provide a cleavable site for cell detachment) were added to the PBMCs. After 30 minutes of incubation at 4°C, cells were washed three times with RPMI/1% fetal calf serum. Supernatant was removed and 4 μl of releasing buffer in 200 μl RPMI/1% fetal calf serum was added to the beads. After 15 minutes of incubation at room temperature, samples were placed in a magnetic device and the released cells were transferred to a different tube. Isolated cells were centrifuged at 2,000 rpm for 2 minutes on glass slides. Double staining microscopy experiments were performed as described previously.

## Results

### Detection of circulating EGFR/CK double-positive tumor cells

**Figure 1:**
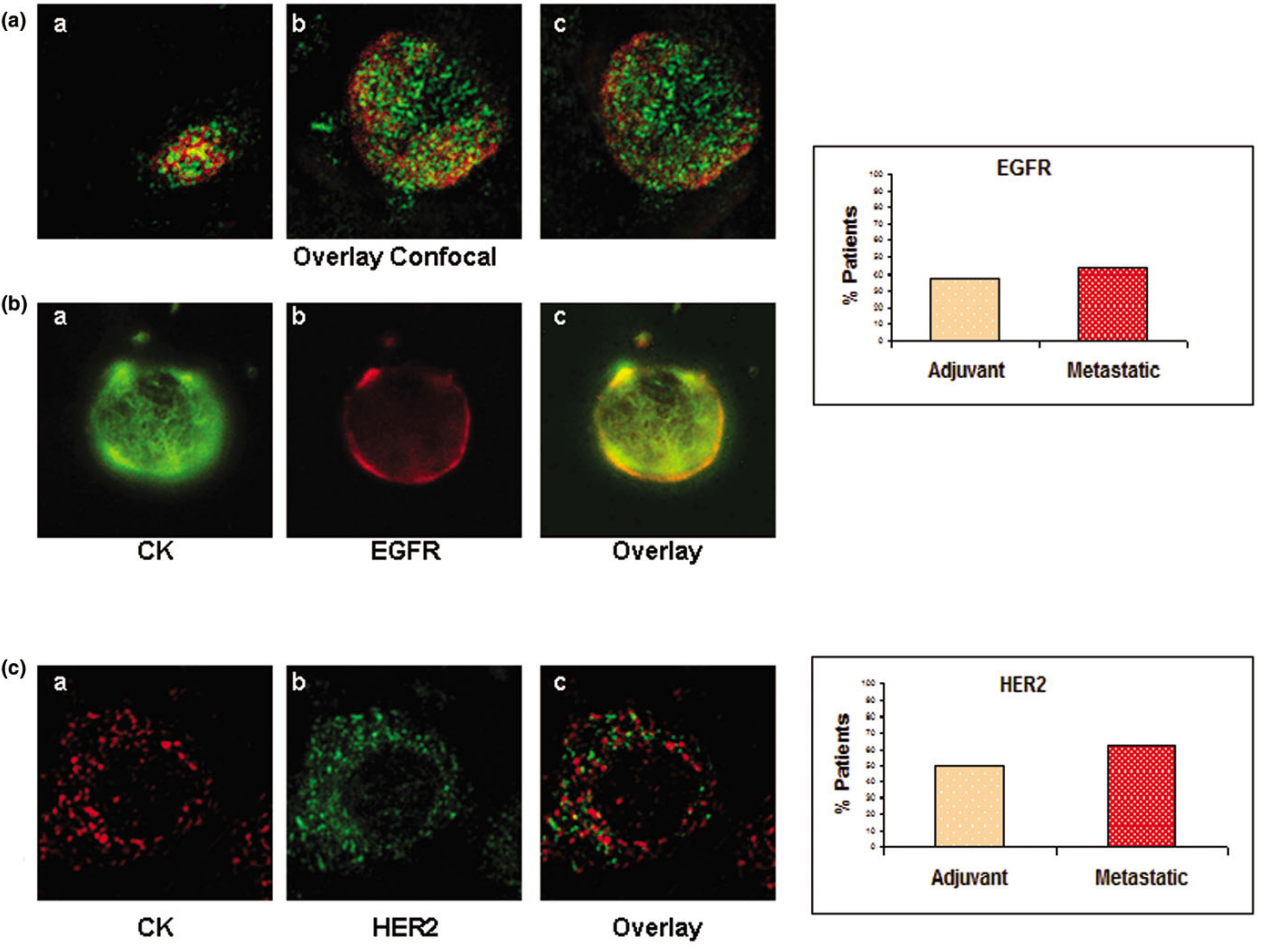
EGFR and HER2 expression in CTCs from breast cancer patients. **(a) **Representative images of confocal laser scanning microscopic sections of PBMC cytospins stained with pancytokeratins A45 B/B3 (green) and EGFR anti-rabbit (red) antibodies, showing consecutive scanning sections from the upper cytoplasmic region toward the basal attachment site in step sizes of 0.8 μm (magnification: 600×). **(b) **Representative images of a cytospin, double stained with monoclonal pancytokeratin (A45-B/B3; green) and polyclonal EGFR anti-rabbit (red) antibodies (magnification: 600×). Also shown is quantification of EGFR and CK co-expression in adjuvant and metastatic CTCs. **(c) **Representative image of cytospin, double stained with polyclonal pancytokeratin and monoclonal HER2 (green) antibodies (magnification: 600×). Quantification of HER2 and CK co-expression in adjuvant and metastatic CTCs. CK, cytokeratin; CTC, circulating tumor cell; EGFR, epidermal growth factor receptor; HER, human epidermal growth factor receptor; PBMC, peripheral blood mononuclear cell.

EGFR-positive CTCs were observed in 38% (six out of 16) and 44% (seven out of 16) patients with early and metastatic breast cancer, respectively (Figure 1; see also Additional file 1). Patient characteristics are presented in Tables 1 and 2. Table 3 presents the frequency of EGFR-positive cells detected per sample. Among a total of 62 CTCs identified in patients with early disease, seven (12%) stained positively for both EGFR and CK, whereas the respective numbers for patients with metastatic disease were 68 (42%) out of 161 cells.

**Table 1:** Patient characteristics: early disease

Characteristic	n (%)
Menopausal status	

Premenopausal	4 (25)

Postmenopausal	12 (75)

Unknown	0 (0)

Tumor size	

pT1	3 (19)

pT2	10 (62)

pT3	3 (19)

Lymph node status	

Node-negative	6(38)

Node-positive	10 (62)

Histologic grade	

Grade 1	1 (6)

Grade 2	11 (69)

Grade 3	4 (25)

ER/PR tumor status	

Positive	11 (69)

Negative	4 (25)

Unknown	1 (6)

HER2 tumor status	

Positive^a^	3 (19)

Negative	12 (75)

Unknown	1 (6)

EGFR tumor status	

Positive	3 (19)

Negative	9 (56)

Unknown	4 (25)

^a^A score +3 or fluorescence *in situ *hybridization positive. EGFR, epidermal growth factor receptor; ER, estrogen receptor; HER, human epidermal growth factor receptor; PR, progesterone receptor.

**Table 2:** Patient characteristics: metastatic disease

Characteristic	n (%)
Menopausal status	

Premenopausal	5 (31)

Postmenopausal	9 (56)

Unknown	2 (13)

Disease sites	

1	1 (6)

2	7 (44)

3	6 (38)

≥ 4	2 (12)

Predominantly visceral disease	

Yes	3 (19)

No	13 (81)

Line of treatment	

0	3 (19)

1	6 (37)

2	3 (19)

≥- 3	4 (25)

ER/PR tumor status	

Positive	11 (69)

Negative	5 (31)

HER2 tumor status	

Positive^a^	5 (31)

Negative	11(69)

EGFR tumor status	

Positive	1(6)

Negative	9 (56)

Unknown	6 (38)

^a^A score +3 or fluorescence *in situ *hybridization positive. EGFR, epidermal growth factor receptor; ER, estrogen receptor; HER, human epidermal growth factor receptor; PR, progesterone receptor.

**Table 3:** Number of EGFR, HER2, pPI3K, and pAkt-positive CTCs in breast cancer patients

Number of CTCs per sample	EGFR			HER2		pPI3K		PAkt
	
	Early disease (n = 16)	Metastatic disease (n = 16)	Early disease (n = 16)	Metastatic disease (n = 16)	Early disease (n = 16)	Metastatic disease (n = 16)	Early disease (n = 16)	Metastatic disease (n = 16)
1	2 (13)	1 (6)	5 (31)	6 (38)	6 (31)	3 (19)	5 (31)	4 (25)

2	4 (25)	1 (6)	1 (6)	2 (13)	5 (31)	2 (13)	4 (25)	3 (19)

3	-	-	-	-	-	4 (25)	1 (6)	2 (13)

4	-	2 (13)	1 (6)	1 (6)	2 (13)		2 (13)	-

≥ 5	-	3 (19)	1 (6)	1 (6)	1 (6)	5 (31)	1 (6)	4 (25)

Values are expressed as number (%). CTC, circulating tumor cell; EGFR, epidermal growth factor receptor; HER, human epidermal growth factor receptor; PI3K, phosphoinositide-3 kinase.

The distribution of EGFR (red staining) and CK (green staining) in CTCs using immunofluorescence microscopy is demonstrated in Figures 1a,b. Sequential sections of the CTCs with confocal laser scanning microscopy confirmed that EGFR staining was mainly membranous and – to a lesser extent – intracellular, whereas CK was mainly located within the cytoplasm (Figure 1a).

As a negative control, PBMCs from 20 healthy female blood donors were also examined for EGFR/CK expression by double staining experiments. There were no cells that could be stained positive for both EGFR and CK in these samples. However, in two samples, EGFR-positive and CK-negative cells were detected. In order to characterize these cells further, double-staining experiments with EGFR and CD45 antibodies were conducted, which revealed positive staining for both proteins, implying a hematopoietic origin for these cells (data not shown).

The EGFR status of primary tumors and CTCs in early and metastatic breast cancer patients is shown in Table 4. In three out of 12 (25%) and four out of 10 (40%) there was a discrepancy in EGFR expression between the primary tumor and CTCs in early and metastatic breast cancer, respectively.

**Table 4:** EGFR and HER2 expression in primary tumor and in CTCs

	Early disease				Metastatic disease			
	
	Primary tumor		CTCs		Primary tumor		CTCs	
	
Patient number	HER2	EGFR	HER2	EGFR	HER2	EGFR	HER2	EGFR
1	-	+	+	+	-	-	+	-

2	+^a^	NA	+	-	+	-	+	-

3	-	-	-	+	+	NA	+	+

4	-	-	+	-	-	-	-	-

5	-	-	+	-	-	-	+	-

6	-	NA	+	+	-	NA	-	-

7	-	+	-	+	+	-	+	-

8	-	-	-	-	-	-	+	-

9	+	-	-	-	-	NA	-	-

10	+	-	+	+	+	-	+	+

11	-	+	-	-	+	NA	-	+

12	-	-	+	-	-	+	-	-

13	-	-	+	-	-	NA	-	+

14	-	-	-	-	-	-	+	+

15	-	NA	-	-	-	NA	+	+

16	NA	NA	-	+	-	-	+	+

^a^Score +3 or fluorescence *in situ *hybridization positive. CTC, circulating tumor cell; EGFR, epidermal growth factor receptor; HER, human epidermal growth factor receptor.

### Detection of circulating HER2/CK double-positive tumor cells

To investigate HER2 expression in CTCs of the same cohort of patients, double-staining experiments were performed using HER2 and CK antibodies. The specificity of these antibodies has been previously reported [26] and no CK-positive/HER2-positive cells could be detected in normal blood donors or in CK-19 mRNA-negative patients with early breast cancer. HER2/CK double-positive cells were identified in CTCs of eight out of 16 patients (50%) with early, and in CTCs from ten out of 16 patients (63%) with metastatic disease (Figure 1c; also see Additional file 1). The frequency of HER2-positive cells detected per sample is presented in Table 3. The intracellular distribution of HER2 using confocal laser scanning microscopy is shown in Figure 1c. Among a total of 51 CTCs identified in patients with early disease, 19 (37%) were CK/HER2-positive, whereas the respective numbers for patients with metastatic disease were 35 out of 103 cells (34%). Three (19%) patients with early and five (31%) patients with metastatic breast cancer had CTCs in their blood expressing both receptors (EGFR and HER2).

As shown in Table 4, there was a discrepancy between the expression of HER2 in primary tumors and CTCs in seven out of 15 (47%) and seven out of 16 (44%) patients with early and metastatic breast cancer, respectively, implying that this is a relatively common phenomenon.

### Evaluation of EGFR and downstream PI-3 and Akt kinases activation in CTCs

**Figure 2:**
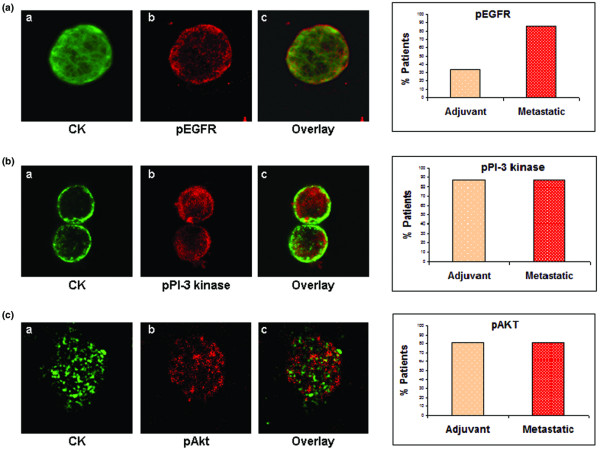
EGFR, PI3K, and Akt activation in CTCs from breast cancer patients. **(a) **Representative micrograph of cytospin, double-stained with monoclonal pancytokeratin (A45-B/B3; green) and polyclonal pEGFR anti-rabbit (red) antibodies (magnification: 600×). Also shown is quantification of pEGFR and CK co-expression in adjuvant and metastatic CTCs. **(b) **Representative image of a cytospin, double stained with monoclonal pancytokeratin (A45-B/B3; green) and polyclonal pPI3K (red) antibodies (magnification: 400×). Also shown is quantification of pPI3K and CK co-expression in adjuvant and metastatic CTCs. **(c) **Representative micrograph of cytospin, double stained with monoclonal pancytokeratin (A45-B/B3; green) and polyclonal pAkt (red) antibodies (magnification: 600 ×). Also shown is quantification of pAkt and CK co-expression in adjuvant and metastatic CTCs. CK, cytokeratin; CTC, circulating tumor cell; EGFR, epidermal growth factor receptor; HER, human epidermal growth factor receptor; PI3K, phosphoinositide-3 kinase.

To investigate whether EGFR is activated in CTCs, double-staining experiments using pEGFR and A45-B/B3 pancytokeratin antibodies were performed. Indeed, pEGFR was observed in patients with EGFR-positive CTCs. The proportion of pEGFR expression in early and metastatic patients was two out of six (33%) and six out of seven (86%), respectively (Figure 2a). Among a total of 20 pEGFR-positive CTCs identified in patients with early disease, three (15%) stained positive for both pEGFR and CK, whereas the respective numbers for patients with metastatic disease were 22 out of 56 cells (39%).

To evaluate whether pro-survival PI3K and Akt signaling kinases operating downstream of EGFR are activated in CTCs, double-staining experiments using CK and pPI3K or pAkt antibodies were performed [16]. It was observed that the great proportion of patients (88%) with both early and metastatic disease harbored CTCs expressing pPI3K (Figure 2b; also see Additional file 1). The frequency of pPI3K-positive CTCs is shown in Table 3. Interestingly, a large proportion of CTCs of each individual patient were pPI3K positive both in the early (median 100%, range 80% to 100%) and the metastatic (median 90%, range 12.50% to 100%) settings.

Similarly, double-staining experiments using CK and pAkt antibodies revealed the presence of pAkt (Figure 2c) in 13 out of 16 patients (81%) both in early and metastatic breast cancer (see Additional file 1). Moreover, a significant proportion of CTCs of each patient were pAkt positive both in early (median 100%, range 16.6% to 100%) and metastatic (median 83%, range 8.70% to 100%) settings. The frequency of pAkt-positive CTCs is presented in Table 3. Double-staining experiments using blood from 10 normal female donors with A45-B/B3 antibody coupled with either pPI3K or pAkt revealed no double-stained cells, implying the absence of CK-positive cells, which express activated signaling kinases in the blood of healthy women.

### Co-expression of EGFR and activated kinases in CTCs

**Figure 3:**
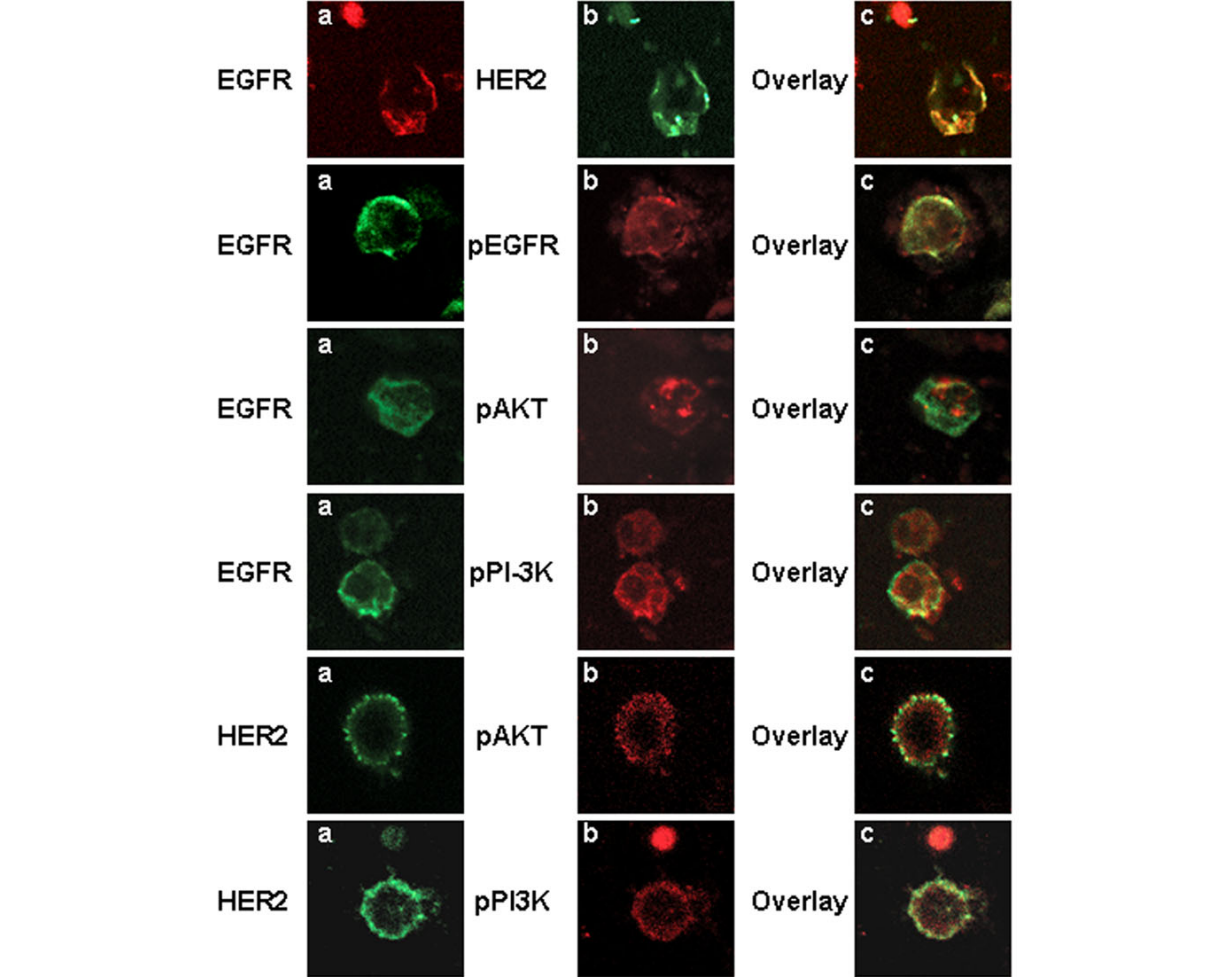
Immumomagnetic separation of CTCs. Representative confocal laser scanning micrographs of cytospins after immunomagnetic separation of CTCs with EpCAM antibody. Cell cytospins were double stained with EGFR antibody and one of the following: HER2, pEGFR, pPI3K, or pAkt antibody. Cytospin were also double stained with HER2 antibody and either pPI3K or pAkt antibodies (magnification: 400×). EGFR is co-expressed with HER2, pEGFR, phospho-PI3K, and pAkt kinase. CTC, circulating tumor cell; EGFR, epidermal growth factor receptor; HER, human epidermal growth factor receptor; PBMC, peripheral blood mononuclear cell; PI3K, phosphoinositide-3 kinase.

To investigate further whether EGFR and HER2 as well as activated PI3K and Akt signaling kinases are co-expressed in CTCs, we performed immunomagnetic separation of CTCs using EpCAM coated beads in three patients whose CTCs were positive for all of the examined molecules. Subsequently, cytospins of the isolated cells were double stained with EGFR and either HER2, pEGFR, pPI3K, or pAkt. As shown in Figure 3, both EGFR and HER2 were co localized on the same cell. Moreover, EGFR was shown to be co-expressed with pEGFR, pPI3K, or pAkt (Figure 3a), implying the presence of an activated pathway in CTCs downstream of EGFR involving PI3K and Akt.

## Discussion

The presence of CTCs in the peripheral blood of patients with breast cancer correlates with poor clinical outcome [4-7]. In recent years, significant effort has been made to achieve specific and sensitive detection of CTCs, which poses significant technical challenges because of the rarity of these cells among an abundance of erythrocytes and leukocytes [9,35,36]. The rarity of CTCs is also a significant limiting factor for their detailed characterization. It is well known that breast cancers exhibit significant heterogeneity and – at least theoretically – the population of CTCs should include the fraction of tumor cells with the biological potential to form metastatic deposits. Therefore, the genomic and phenotypic analysis of CTCs is of paramount importance for patients with breast cancer, because it provides an insight into the metastatic properties of the primary tumor and permits the exploitation of targeted therapies that are more likely to be effective. A CK-positive cell can be characterized as a CTC if cytomorphological criteria described by Meng and coworkers [33] are satisfied. Furthermore, we and others have previously demonstrated the malignant origin of CK-positive cells, because fluorescence *in situ *hybridization (FISH) analysis identified HER2 gene amplification and aneusomy [10,12].

EGFR is frequently over-expressed in breast cancers [19,20], and EGFR signaling is correlated with poor patient outcome [22,23]. In the present study, by using immunofluorescence microscopy, we detected EGFR-positive CTCs in 38% and 44% of patients with early and metastatic disease, respectively, harboring CTCs in their blood. In accordance with these findings, EGFR mRNA expression has previously been reported in the blood of 22% to 48% of patients with metastatic breast cancer [37,38]. However, it should be noted that molecular techniques suffer from a relative lack of specificity, because illegitimate transcription of the EGFR gene in leukocytes cannot be excluded [39]. Conversely, cytological techniques permit evaluation of cell morphology and identification of those cells that have tumor-like appearance. In accordance with this assumption, in the present study we identified EGFR expressing hematopoetic cells in 10% of samples from healthy donors.

Despite the relatively small number of patients for whom the primary tumor was available for EGFR evaluation, it was evident that an EGFR-negative primary tumor does not preclude the presence of EGFR-expressing CTCs. Moreover, a higher proportion of the evaluated CTCs was stained positive for EGFR in patients with metastatic than in patients with early breast cancer. This observation suggests that EGFR is expressed in a predominant clone of cells that contribute to disease progression. Conversely, HER2 expression in the total number of CTCs in early and metastatic disease was similar. This could probably be explained by the observation that human breast cancer is often characterized by a gradual progression to an estrogen-independent, EGFR-positive, and highly metastatic phenotype [40].

In a previous report [26], HER2 expression was reported in cells circulating in the blood of patients with early breast cancer. In another study [15], the presence of HER2-expressing CTCs was found to be suggestive of poor disease-free and overall survival in stage I to III breast cancer. HER2/CK-positive cells were recently characterized as tumor cells using FISH analysis [11]. In the present study, double-staining experiments using pancytokeratin and HER2 antibodies revealed that CK-positive CTCs also co-express HER2. The 50% expression rate in patients with early disease shown here is almost identical to the 48.6% of HER2-positive CTCs in patients with primary breast cancer reported recently by Wulfing and coworkers [15]. It is interesting to note that, in accordance with the above mentioned study [15], we observed a discrepancy between the detection of HER2-positive CTCs and the HER2 staining score of the corresponding primary tumor (Table 4). HER2 positivity in the primary tumor was assessed in accordance with the 2007 criteria proposed by Wolff and coworkers [41], and FISH evaluation was performed in patients with immunohistochemical staining score 2. This discrepancy was also observed in the EGFR expression pattern (Table 4). This could be related to the different methods used for the evaluation of these receptors in the primary tumors and/or the heterogeneity of CTCs. Interestingly, by using immunomagnetic separation of CTCs in three patients, co-localization of both receptors on same cells was demonstrated, thus suggesting that an EGFR and HER2 crosstalk may be operating [42].

Additional experiments revealed that pEGFR is also expressed on the CTCs of both early and metastatic breast cancer patients. The presence of pEGFR on CTCs further supports the hypothesis that EGFR plays a functional role in the biology of these cells. Indeed, EGFR activation was demonstrated in a higher proportion of patients with metastatic disease compared with patients with early disease. Moreover, a higher proportion of the total number of EGFR-expressing CTCs was activated in advanced compared with early disease. Although the small number of patients examined precludes firm conclusions, this finding further suggests that EGFR signaling may be involved in breast cancer progression.

PI3K and Akt kinases operate downstream of EGFR and HER2 in cancer cells, transmitting signals that regulate cell survival and cell migration [16,43-45]. In a previous report [26], pPI3K was shown to be expressed on the CTCs of patients with early breast cancer. Here we report that pPI3K and pAkt were identified in a significant proportion of CTCs from patients with early and metastatic disease. Their co-localization with EGFR is suggestive of an active pathway involving PI3K and Akt that is initiated upon EGFR activation. Moreover, a statistically significant correlation (*P *= 0.038) between the number of CTCs expressing pPI3K and pAkt in metastatic patients was observed, suggesting the existence of an active pathway in these cells; conversely, the same correlation was not observed in patients with early breast cancer. This could be due to the heterogeneity of CTCs in these patients [13]. Because the aim of the present study was to evaluate the expression of EGFR, HER2, pAkt and pPI3K in CTCs, the presented data do not have the statistical power to establish clinical correlations because of the small number of enrolled patients. The clinical significance of EGFR, HER2, pAkt, and pPI3K expression in CTCs should be investigated in large prospective clinical trials.

## Conclusion

This is the first report to demonstrate expression of activated EGFR and Akt in CTCs in both adjuvant and metastatic settings. The co-expression of HER2 receptor could confer a more aggressive phenotype on EGFR-positive CTCs [46,47]. Our observations suggest that EGFR-triggered and/or HER2-triggered PI3K/Akt activation may be involved in the regulation of the malignant and metastatic potential of CTCs. Therefore, these molecules could serve as targets for the elimination of micrometastatic disease. In view of the limited capacity of standard systemic treatments to control or eliminate CTCs [48], the data presented in the present study could have important therapeutic implications for novel targeted treatment strategies against micrometastatic disease.

## Abbreviations

CK: cytokeratin; CTC: circulating tumor cell; EGFR: epidermal growth factor receptor; FISH: fluorescence *in situ *hybridization; HER: human epidermal growth factor receptor; PBMC: peripheral blood mononuclear cell; PI3K: phosphoinositide-3 kinase.

## Competing interests

The authors declare that they have no competing interests.

## Authors' contributions

GK participated in the design and coordination of the study. She performed the immunofluoresence experiments as well as the immunomagnetic separations and the CTC cultures. She also drafted the manuscript. SA participated in the design coordination of the study and drafted the manuscript. AK collected all the clinicopathological characteristics of the patients. CS participated in the coordination of the study and helped to draft the manuscript. DM participated in the design and coordination of the study, and helped to draft the manuscript. VG provided general support and participated in study design.

## Supplementary Material

Additional file 1 File listing the expression levels of CK, EGFR, pEGFR, HER2, pPI3K, and pAkt in CTCs of breast cancer patients. Click here for file
