# Supplementary material for: Phosphorylated EGFR and PI3K/Akt signaling kinases are expressed in circulating tumor cells of breast cancer patients
Source: Breast Cancer Res. 2008 Sep 29;10(5):R80. doi: 10.1186/bcr2149 (PMC2614515; doi:10.1186/bcr2149)
Supplement: Additional file 1 — File listing the expression levels of CK, EGFR, pEGFR, HER2, pPI3K, and pAkt in CTCs of breast cancer patients. [file bcr2149-S1.doc]

**Supplementary Table 1 .** EGFR, HER2, pEGFR, pAkt and pPI-3K expression in CTCs of breast cancer patients

|  | **Adjuvant** | **Metastatic** |
| --- | --- | --- |
| **CK positive** | 16 (100%) | 16 (100%) |
| **EGFR positive** | 6 (38%) | 7 (44%) |
| **pEGFR positive** | 2 (13%) | 6 (38%) |
| **HER2 positive** | 8 (50%) | 10 (63%) |
| **pPI-3K positive** | 14 (88%) | 14 (88%) |
| **pAkt positive** | 13 (81%) | 13 (81%) |
